# Creativity and aesthetic evaluation. Two proposals to improve the model of aesthetic dis/fluency

**DOI:** 10.3389/fpsyg.2014.01520

**Published:** 2015-01-08

**Authors:** Gianluca Consoli

**Affiliations:** Department of Philosophy, University of Rome Tor VergataRome, Italy

**Keywords:** perception, aesthetic pleasure, dis/fluency, feelings of knowing, mood

In recent years an innovative frame concerning the link between perception and appreciation of art has been proposed. This frame has been clearly articulated by Van de Cruys and Wagerman (2011). An increasing experimental evidence significantly corroborates it. Here we can refer to the data collected by Muth et al. (2012), Muth and Carbon (2013) and Carbon and Hesslinger (2014).

According to Van de Cruys and Wagerman (2011), the central idea is that more often than not great artworks do not allow an immediate and easy recognition—so that the more fluent the processing, the higher the appreciation (Bullot and Reber, 2013). On the contrary, they are creative and innovative, challenging and surprising. They inhibit ordinary perceptual routines, violate predictions, involve disorder, disorganization, disharmony, ambiguity, contradictions, indeterminacy, uncertainty, strangeness, and so on. However, viewers—with a sufficient degree of expertise (Leder et al., 2014)—often succeed in establishing a new predictable pattern on a different level. This transition from an initial state of uncertainty, associated with unpleasant and negative affect, to a subsequent state of increased predictability and fluency is highly rewarding. So, aesthetic appreciation is not static, but dynamic. As it is suggested by the study of Carbon and Hesslinger (2014), it strongly depends on the quality of elaboration in terms of extended, active, deep processing. Besides increasing familiarity, this elaboration enables insights into the meanings of artworks and allows viewers to identify new patterns.

For the first time recent experiments concerning the aesthetic appreciation empirically demonstrate the deep relationship between perceptual insights and aesthetic pleasure.

In the first study realized by Muth et al. (2012), photographs of cubist artworks by Picasso, Braque, and Gris were shown to participants without expertise in cubist art. The study was structured in two blocks, each showing the stimuli in a randomized order. During the first block, subjects had to rate the pictures on liking. During the second block, subjects rated how well they could detect objects within the artwork. All ratings were chosen from a 7-point-Likert-scale from 1 (“not at all”) to 7 (“very”). Data across participant revealed a strong relationship between the detectability of objects and liking, confirming that also in aesthetic perception form recognition is closely related to appreciation.

In the second study realized by Muth and Carbon (2013), two-tone images either containing a hidden form (i.e., a face) or not were repeatedly presented for half a second to participants. Stimuli were shown in a randomized order block-wise 13 times. The tasks alternated block-wise between choosing from a 7-point scale from 1 (“not at all”) to 7 (“very good”) how much one liked the picture and a detecting block. The latter comprised two ratings on a 1 plus 7-point scale (0: “no face recognized”; 7: “very clear”). Insight was defined by the highest gain in clearness between two subsequent blocks per participant and stimulus. All liking ratings per participant and block were then shifted in regard to their temporal occurrence relative to the insight block. Data clearly demonstrated that liking only significantly increased after having an insight; the intensity of insight, defined as degrees of clearness ratings, showed direct influences on the degrees of liking.

In my perspective, the important frame of the aesthetic dis/fluency can be both refined and extended so that it can gain an higher explanatory power. I propose two theoretical hypothesis. They are not derived from experimental evidence directly concerning aesthetic experience. However, these hypothesis are suggested by what we already know about relevant mechanisms and functions of the so-called “affective mind” (Hatano et al., 2000)—an interdisciplinary field of study in cognitive sciences dedicated to “hot” phenomena, such as affect, bodily sensations, pleasure and displeasure, emotions, mood, and so on.

First, we can refine the conception of the (“early”) aesthetic pleasure associated with a single insight into a determined pattern. From this point of view, it is very plausible that this kind of aesthetic pleasure does not represent only a phenomenal signal concerning the specific, already achieved, Gestalt formation. On the contrary, it signals at the same time that there is more, that other processes of integration are available. It indicates that the artwork enacts a higher potential of integration, that it enables a complex network of cues, associations, and meanings allowing further cycles of explorations—precisely what happens with the Cubist paintings. In this way the aesthetic pleasure contributes to guiding the subsequent exploration of the artwork, facilitating the more promising lines of scrutiny, the positively rewarded ones. In this perspective, the aesthetic pleasure is undoubtedly a function of the quality of elaboration, but it is different in kind from the affective reward that follows problem solving. It does not constitute only a *post factum* subjective reaction of satisfaction, a passive gratification merely prompted by the identification of a new recognizable pattern. The aesthetic pleasure is a crucial component of the aesthetic processing dynamics because it provides an essential anticipatory function.

A next step in empirical research on the perception of art might be the study of this anticipatory function. However, in virtue of this property the aesthetic pleasure seems to be very similar to the so-called “feelings of knowing” (or “knowing feelings”). This kind of feelings are subjective experiences accompanying thought. They are automatically elicited by internal and experiential cues associated with the ongoing processing dynamics. Individuals tacitly consult them as signals concerning the state of their knowledge and implicitly produce intuitive metajudgments in the form: “I feel that I know/retrieve/learn x” (Koriat, 2000; Schwarz and Clore, 2007). Aesthetic pleasure and feelings of knowing are both high-order phenomenal signals grounded in and function of the first-order processing experience of the subjects. They are both not conceptual, not analytical, not verbal, not systematic, and not deliberate cues that accompany elaboration and influence it, mediating the allocation of attention, time, and effort. With a crucial difference: the feelings of knowing usually are determined by the properties of the availability heuristics (the immediacy of recognition, the direct accessibility of pertinent information, the ease with which the information comes to mind, the familiarity of the stimulus, the fluency of the processing). On the contrary, when it is prompted by great artworks, the aesthetic pleasure is often determined a dis/fluent processing.

Secondly, we can extend the conception of the (“late”) aesthetic pleasure associated with multiple cycles of insights. From this point of view, great artworks provide an optimal amount of (un)predictability, neither too much (disturbing), nor too little (annoying). This amount of (un)predictability is embodied in a pool of micro-rewards hidden in the artwork (Van de Cruys and Wagerman, 2011). So the aesthetic appreciation does not increase in a linear and progressive fashion: new challenges will initially involve unpleasant experiences. However, according to the experimental evidence collected about the phenomenon of mood—an overall and pervasive affective state, produced by a set of implicit and tacit appraisals (Forgas, 2007)—we might also expect that, during the interpretation of a great artwork, when the viewer has already solved a certain quantity of difficulties, he begins to feel good. Even if the process of interpretation is still open and he has to face other challenges, we might expect that he begins to have a positive mood, determined by the previous series of insights and rewards. Enhancing this diffuse and enduring positive affect, great artworks can support and stimulate viewers' further explorations even when the degree of discrepancy is significantly high or discrepancy remains temporarily unsolved.

In line with this proposal, it is possible that the perception of art is associated with two different, intertwined kinds of aesthetic pleasure. The early aesthetic pleasure, correlated to the specific challenges of the dis/fluency process and active since the early insights. The late aesthetic pleasure, correlated to the dis/fluency process as such and active only after a certain amount of insights. Another step in future research in experimental aesthetics might be the study of the late aesthetic pleasure and its interaction with the early aesthetic pleasure.

## Conflict of interest statement

The author declares that the research was conducted in the absence of any commercial or financial relationships that could be construed as a potential conflict of interest.
